# Manufacturing Polypropylene (PP)/Waste EPDM Thermoplastic Elastomers Using Ultrasonically Aided Twin-Screw Extrusion

**DOI:** 10.3390/polym13020259

**Published:** 2021-01-14

**Authors:** Hui Dong, Jing Zhong, Avraam I. Isayev

**Affiliations:** Department of Polymer Engineering, The University of Akron, Akron, OH 44325-0301, USA; hd16@zips.uakron.edu (H.D.); jz56@zips.uakron.edu (J.Z.)

**Keywords:** ultrasound, extrusion, rheology, polypropylene, EPDM, elastomer

## Abstract

The compounding of waste EPDM from postindustrial scrap with polypropylene (PP) is a possible way to manufacture thermoplastic elastomers to solve a significant environmental problem. Accordingly, the present study considers the one-step (OS), two-step (TS), and dynamic revulcanization (DR) compounding methods for the manufacturing of PP/EPDM blends at different ratios of components with the aid of an ultrasonic twin-screw extruder (TSE) at various ultrasonic amplitudes. In the OS method, PP and waste EPDM particles were directly compounded using TSE with and without ultrasonic treatment. In the TS and DR methods, the waste EPDM particles were fed into the TSE and devulcanized without and with ultrasonic treatment. Then, in the TS method the devulcanized EPDM was compounded with PP using TSE without the imposition of ultrasound. In the DR method, the devulcanized EPDM after compounding with curatives was mixed with PP and dynamically revulcanized without the imposition of ultrasound in TSE. The die pressure during compounding was recorded and correlated with the rheological properties of compounds. The mechanical properties of the PP/EPDM blends obtained in the OS and TS methods did not show any improvement with ultrasonic treatment. In the DR method, all the PP/EPDM blends showed a significant increase in the tensile strength and elongation with ultrasonic amplitude and a slight decrease in the Young’s modulus. In particular, a tensile strength of 30 MPa and an elongation at break of 400% were achieved at an ultrasonic amplitude of 13 μm for the PP/EPDM blend at a ratio of 75/25. The complex viscosity, storage, and loss moduli of dynamically revulcanized PP/EPDM blends increased with the ultrasonic amplitude while the loss tangent decreased. At the same time, the results for the blends obtained by the OS and TS methods showed an opposite trend in the dynamic property behavior with the ultrasonic amplitude. Optical micrographs indicated that the blends obtained by the DR method at an ultrasonic treatment at 13 μm showed the lowest sizes of dispersed revulcanized EPDM particles in the PP matrix, leading to the excellent performance of these thermoplastic elastomers.

## 1. Introduction

The manufacturing of thermoplastic elastomers (TPEs) based on blends of plastics with rubbers is cost-effective and practically valuable. TPEs are materials that exhibit both thermoplastic and elastomeric properties. TPEs have advantages such as ease of processing as well as the possibility to tailor properties by changing the ratio of the components. They also display disadvantages, including softening and losing their rubbery behavior at elevated temperatures [1].

Many TPEs are obtained by the dynamic vulcanization. During this process, elastomer particles are vulcanized and dispersed in the thermoplastic matrix during vulcanization. The properties of the blends are improved, with a reduction in the size of the elastomer particles. The process was first introduced in 1962 [2]. The first TPEs based on a crosslinked rubber-thermoplastic composition were derived in 1973 by partially crosslinking the EPDM with peroxide in a PP matrix [3]. Important enhancements in the properties of these blends were achieved in 1978 by fully vulcanizing the rubber phase while maintaining the processability of the thermoplastics [4]. In 1982, these blends were further improved by the use of phenolic resin as a curative, improving the rubber-like properties and the processing features [5]. Possible applications of thermoplastic elastomers and thermoplastic vulcanizates are flexible diaphragms, bumpers, seals, plugs, and wire and cable insulation. Due to these applications, research on novel PP/EPDM thermoplastic vulcanizates remains active nowadays, as is evident from the recent publications on the topic [6,7,8,9,10].

At the beginning of this century, a novel method was proposed to enhance the properties of plastic/plastic, plastic/rubber, and rubber/rubber blends by means of ultrasonic compatibilization [11,12,13,14]. It was found that during mixing with the aid of ultrasound, the compatibilization and interfacial adhesion between diverse phases are improved. Research on the in situ compatibilization of PP/EPDM blends using ultrasound-aided extrusion was also conducted [15]. The mechanical properties of the compression-molded samples of TPEs made from ultrasonically treated PP/EPDM were improved due to the formation of a copolymer during ultrasonic treatment. Ultrasound was also successfully used to prepare the TPE consisting of PP and virgin EPDM [16]. In another study [17], maleic anhydride grafted PP was compounded with waste EPDM powder of sizes between 5 and 20 μm using a twin screw extruder with an ultrasonic horn at the exit. The study showed that ultrasonic treatment led to improved performance properties. However, random co-PP/waste EPDM blends showed the deterioration of the properties after ultrasonic treatment.

Therefore, it is important to search new way to develop a process for manufacturing TPEs from blends of PP/waste EPDM powder. In the current study, this was accomplished by using the ultrasonic twin screw extruder (TSE) recently developed in our laboratory, in which the ultrasonic horn was installed in the barrel. The effect of ultrasonic treatment on the mechanical and rheological properties of the PP/waste EPDM blends prepared using several methods was studied using this ultrasonic TSE. A method to obtain TPEs based on PP/waste EPDM powder with a good mechanical performance was proposed.

## 2. Experimental

### 2.1. Materials

The PP used for preparing PP/waste EPDM blends was metallocene-based polypropylene PP3825 supplied by the Exxon Chemical Company. Its melt flow rate is 32g/10min and its molecular weight is 144,800 [18]. Waste EPDM rubber from the post-industrial scrap of 40 meshes (MD-184-EPDM) manufactured by Lehigh Technologies, Inc. (Tucker, GA, USA), was used.

#### 2.1.1. Ultrasonic Twin-Screw Extruder

**Figure 1:**
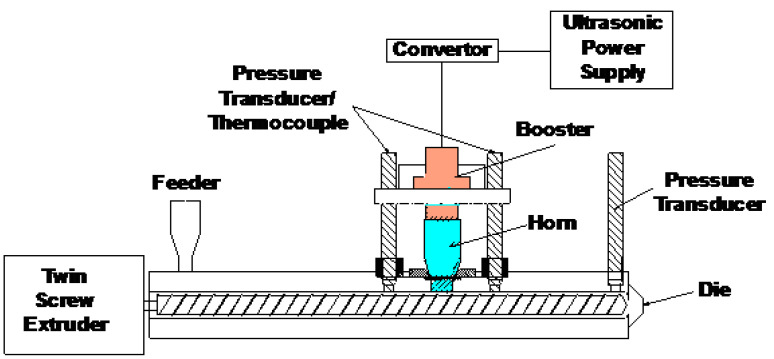
Schematic of the ultrasonic twin screw extruder.

**Figure 2:**
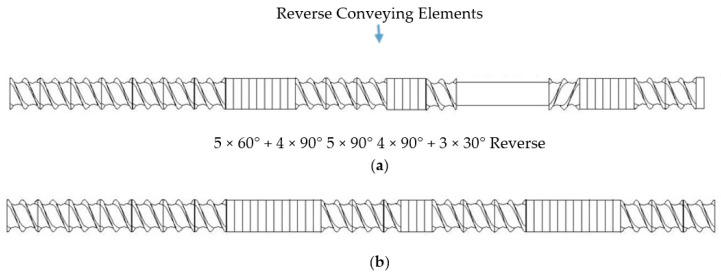
Schematics of the ultrasonic (**a**) and mixing (**b**) screws. The flow direction is from left to right.

Devulcanization and compounding was carried out using an ultrasonic co-rotating TSE (Prism USALAB 16, Thermo Electron Co., Waltham, MA, USA). A schematic of this ultrasonic TSE is shown in Figure 1. Two screws with a diameter of 16 mm were used to convey and mix the EPDM rubber with PP. Ultrasonic waves with a frequency of 40 kHz were applied to the EPDM rubber by a horn mounted in the barrel. The horn has a 28 mm × 28 mm square cross-section and was connected to a booster and a converter. The converter was driven by a Branson 2000bdc power supply (Branson Ultrasonic Co., Danbury, CT, USA). The gap between the horn and screws was 2.5 mm, and the volume of the ultrasonic treatment zone was 1.9 cm^3^. The circular die of the extruder had a diameter of 4 mm and a length of 11 mm. The ultrasonic horn was cooled by water at 45 °C coming from a thermostat (GP-100, NESLAB Instruments Inc., Newington, NH, USA). Additionally, the converter was cooled by compressed air. Figure 2 shows the schematics of two screw designs Figure 2a,b. The screw design (Figure 2a) contained conveying and kneading elements and cylindrical elements in the ultrasonic treatment zone. The screw design (Figure 2b) was a mixing design containing conveying and kneading elements without the ultrasonic treatment zone.

#### 2.1.2. Preparation of PP/Waste EPDM TPEs

Three different methods to manufacture PP/waste EPDM thermoplastic elastomers were used. The first method is called the one-step (OS) method. In this method, the PP pellets were fed into the hopper of the TSE using a feeder (K-Tron Soder, Houston, TX, USA), and the waste EPDM was also fed into the same hopper using a high-precision twin-screw feeder (MT-2, Brabender Technologie GmbH & Co. KG, Duisburg, Germany). The screw design shown in Figure 2a was used without and with ultrasonic treatment at amplitudes of 7.5, 10, and 13 μm. The total flow rate was 8 g/min. The screw speed was 200 rpm, and the zone temperatures from the entrance of the extruder to the die were 150/180/190/190/190/190 °C. The extrudates from extruders were cooled, dried, and pelletized using a pelletizer (Scheer Bay Company, Bay City, MI, USA). The second method is called the two-step (TS) method. In this method, waste EPDM was fed into an extruder equipped with the screw design depicted in Figure 2a at a flow rate of 8 g/min and devulcanized without and with ultrasonic treatment at amplitudes of 7.5, 10, and 13 μm. Then, PP/devulcanized EPDM blends of 75/25, 50/50, and 25/75 compositions were physically mixed and fed to the TSE using the screw design depicted in Figure 2b at a flow rate of 8 g/min without the ultrasonic treatment. The screw speed and zone temperatures were same as in the OS method. The third method is called the dynamic revulcanization (DR) method. In this method, waste EPDM was fed into the extruder using the screw design depicted in Figure 2a at a flow rate of 8 g/min and devulcanized without and with ultrasonic treatment at amplitudes of 7.5, 10, and 13 μm. After extrusion, the devulcanized EPDM rubber was compounded with curatives using a two-roll mill (Reliable Rubber & Plastic Machinery Co., North Bergen, NJ, USA) with a gap size of 5 mm. A rotor speed of 20 rpm and a cooling water temperature of 40 °C were used. Then, it was crushed into particles using a grinder (Weima). The compounding recipe was as follows: 100 phr devulcanized EPDM rubber, 1 phr sulfur, 2 phr zinc oxide, 1 phr stearic acid, 0.75 phr TMTD and 0.375 phr MBT. All the curatives were supplied by Akrochem Corporation (Akron, OH, USA). Finally, PP/devulcanized EPDM blends of 75/25, 50/50, and 25/75 compositions were physically mixed and then fed to the TSE using the screw design depicted in Figure 2b at a flow rate of 8 g/min without the imposition of ultrasound. The screw speed and zone temperatures were same as in the OS method. The extrudates were then cooled, dried, and finally pelletized using a pelletizer (Scheer Bay Company, Bay City, MI, USA). The size of these particles was about 2 mm.

### 2.2. Characterization Methods

#### 2.2.1. Tensile Test

PP/EPDM tensile bars at ratios of 75/25 and 50/50 were prepared using a mini-jet injection molding machine (DSM Research B.V). A cylinder temperature of 200 °C and a mold temperature of 60 °C at a pressure of 40 MPa were used. The PP/EPDM blends at a ratio of 25/75 could not be prepared by injection molding due to their high viscosity. Thus, tensile samples of these PP/EPDM blends were prepared by a compression molding press (Carver Inc., Wabash, IN) at 180 °C under a pressure of 13.8 MPa with a mold of dimensions 90 mm × 60 mm × 1.5 mm.

The tensile tests of pure PP and PP/EPDM blends were conducted by an Instron Tensile Tester 5567 at room temperature at an elongation rate of 50 mm/min. An extensometer was not used. The tests were operated according to the ASTM D638 standard.

#### 2.2.2. Rheological Tests

A small amplitude oscillatory shear (SAOS) test of the pure PP and PP/EPDM blends at ratios of 75/25 and 50/50 at a temperature of 180 °C was conducted by using a stress-controlled Discover Hybrid Rheometer (DHR-2, TA Instruments, New Castle, DE, USA) equipped with 25 mm parallel plates. Specimens of pure PP and PP/EPDM blends were molded at 180 °C under a pressure of 25 MPa for 5 min using a compression molding press (Carver Inc.). The samples had a diameter of 25 mm and thickness of 2 mm. For PP/EPDM blends at a ratio of 75/25, the frequency sweep was in the range of 0.1 to 200 rad/s at a stress amplitude from 4 to 40 Pa. It was confirmed that the stress amplitudes were within the linear region. The rheological properties of the PP/EPDM blends at ratio of 25/75 cannot be tested by DHR-2 due to the wall slip. The rheological properties of these blends were measured using the Advanced Polymer Analyzer (APA 2000, Alpha Technologies, Akron, OH, USA) at 180 °C and a strain amplitude of 4.2% within a frequency range from 0.01 to 200 rad/s.

#### 2.2.3. Morphological Tests

The sizes of the original EPDM powder and the phase morphology of the PP/EPDM blends were observed using an optical transmission microscopy (Laborlux 12 POL S, Leitz Ltd., Midland, ON, Canada). Thin films of about 20 μm were cut using a microtome (Model 820, Reicher-Jung GmbH, Nussloch, Germany) from an injection molded dumbell sample. The images were captured by a camera and then analyzed using the ImageJ software.

## 3. Results and Discussion

### 3.1. Die Pressure

**Figure 3:**
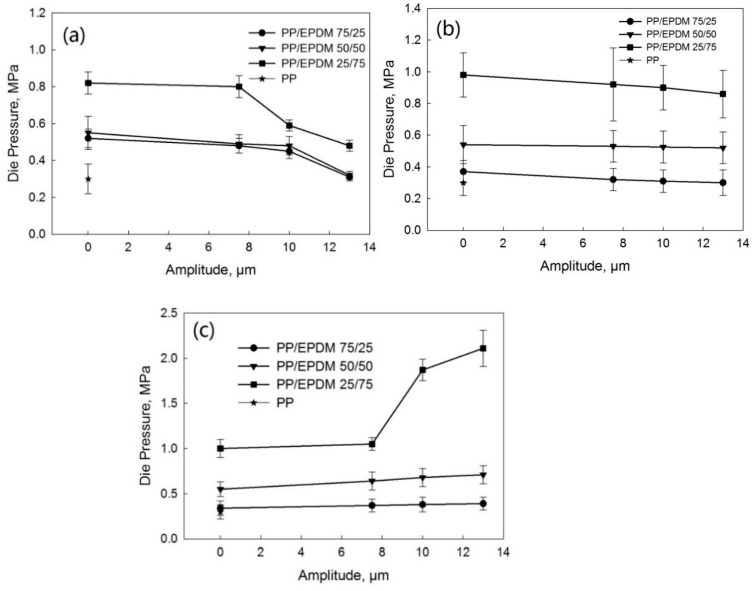
Die pressure as a function of the ultrasonic amplitude of the PP/EPDM blends prepared by the OS (**a**), TS (**b**), and DR (**c**) methods. Die pressure for pure PP is also indicated.

The pressure before the die during the twin-screw extrusion of PP/waste EPDM blends from TSE as a function of the ultrasonic amplitude at various PP/EPDM ratios is presented Figure 3a–c for the OS, TS, and DR methods, respectively. For the OS and TS methods, the die pressure decreased with the increasing concentration of PP due to the lower viscosity of PP than that of EPDM rubber. The die pressure also reduced with an increase in the ultrasonic amplitude, indicating the better processability of the melts under ultrasonic treatment. The decrease in the die pressure with amplitude in the OS method was due to the degradation of PP and the devulcanization of EPDM caused by the acoustic cavitation. For the TS method Figure 3b, the devulcanization of waste EPDM led to a reduction in the complex viscosity, which caused a reduction in the die pressure. However, for the DR method shown in Figure 3c, the die pressure increased with an increase in the ultrasonic amplitude. During the dynamic revulcanization, the chemical network generated by the curing of the EPDM phase in the blends led to the higher complex viscosity of the blends, as shown below.

### 3.2. Dynamic Properties

**Figure 4:**
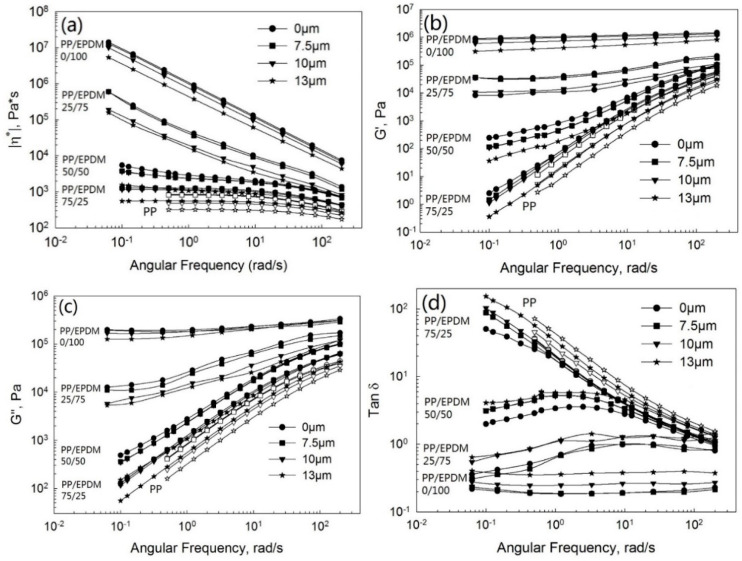
Complex viscosity (**a**), storage modulus (**b**), loss modulus (**c**), and loss tangent (**d**) as a function of the frequency for the pure PP, devulcanized EPDM, and PP/EPDM blends prepared by the OS method.

Figure 4 shows the complex viscosity (Figure 4a), storage (Figure 4b) and loss (Figure 4c) moduli, and tangent loss (Figure 4d) as a function of the frequency at various ultrasonic amplitudes for pure PP, devulcanized EPDM, and PP/EPDM blends at ratios of 75/25, 50/50, and 25/75 from TSE using the OS method. Firstly, the complex viscosity of the blends increased tremendously with the increasing EPDM concentration. According to the earlier study [11], this occurs due to the higher viscosity of EPDM rubber than that of PP, leading to the higher viscosity of PP/EPDM blends. This is also in accordance with the higher die pressure of blends, as shown in Figure 3a. Secondly, it is seen that the complex viscosity of PP decreased the with increasing ultrasonic amplitude due to the degradation of PP. Moreover, it can be seen that the complex viscosity of PP/EPDM blends decreased with the increasing ultrasonic amplitude at different weight ratios of PP/EPDM blends. The complex viscosity was affected by two factors: the devulcanization of EPDM and the degradation of PP under ultrasonic treatment. Specifically, the complex viscosity of ultrasonically treated PP/EPDM blends at a ratio of 75/25 and at an amplitude of 13 μm decreased significantly. It was even lower than the complex viscosity of the untreated PP. The storage (Figure 4b) and loss (Figure 4c) moduli decreased dramatically at an amplitude of 13 μm at all ratios of PP/EPDM blends. The loss tangent (Figure 4d) increased with ultrasonic amplitude, with the highest value being at an amplitude of 13 μm. These observations were due to the fact that the higher ultrasonic amplitude caused a higher level of devulcanization of EPDM and more degradation of PP. Both the ultrasonic devulcanization of EPDM and PP degradation are considered to be induced by acoustic cavitation [19,20].

**Figure 5:**
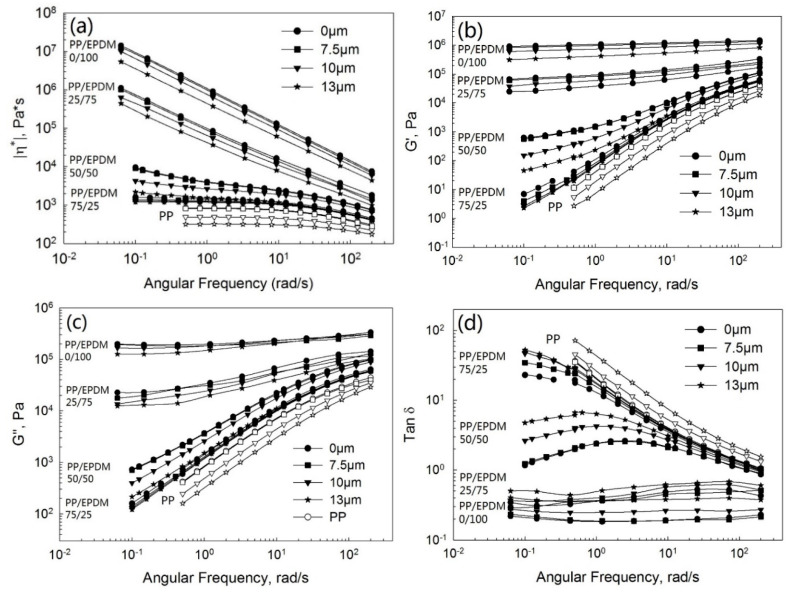
Complex viscosity (**a**), storage moduli (**b**), loss moduli (**c**), and loss tangent (**d**) as a function of the frequency for the pure PP, devulcanized EPDM, and PP/EPDM blends prepared by the TS method.

Figure 5 depicts the complex viscosity (Figure 5a), storage (Figure 5b) and loss (Figure 5c) moduli, and tangent loss (Figure 5d) as a function of the frequency at various ultrasonic amplitudes for pure the PP, pure EPDM, and PP/EPDM blends of the ratios of 75/25, 50/50, and 25/75 from TSE using the TS method. Compared to the OS method, the complex viscosity of the blends increased tremendously with the increasing EPDM concentration due to the higher viscosity of EPDM rubber than that of pure PP, leading to the higher viscosity of PP/EPDM blends. This is also in accordance with the die pressure shown in Figure 3b. Additionally, it can be seen that the complex viscosity of the PP/EPDM blends decreased with the increasing ultrasonic amplitude at various ratios of PP/EPDM blends. However, at this time the complex viscosity of the blends is just dependent on the extent of the devulcanization level of EPDM, because PP was not treated by ultrasound. Besides this, the storage (Figure 5b) and loss (Figure 5c) moduli decreased dramatically at an amplitude of 13 μm. The loss tangent (Figure 5d) shows the highest value at an amplitude of 13 μm. These observations are due to the fact that, at such a high ultrasonic amplitude, more devulcanization of EPDM occurs, leading to a lower value of the gel fraction of devulcanized EPDM rubber [16,17].

**Figure 6:**
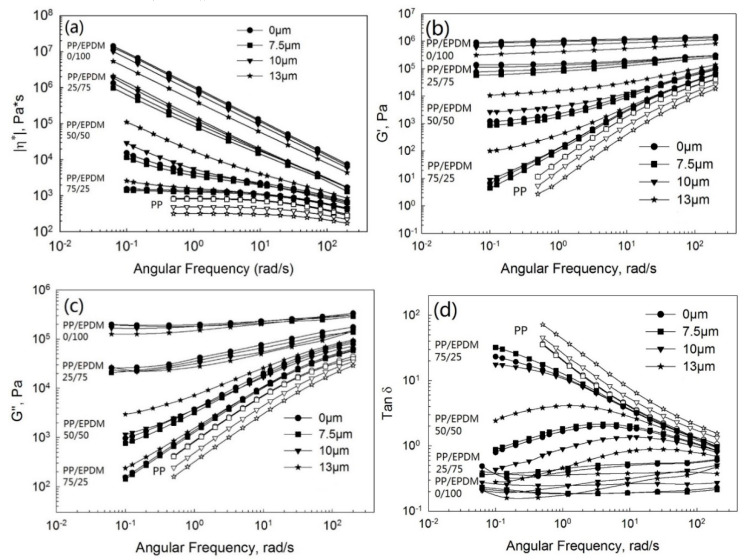
Complex viscosity (**a**), storage modulus (**b**), loss modulus (**c**), and loss tangent (**d**) as a function of the frequency at various ultrasonic amplitudes for the pure PP, devulcanized EPDM, and PP/EPDM blends prepared by the DR method.

Figure 6 shows the complex viscosity (Figure 6a), storage (Figure 6b) and loss (Figure 6c) moduli, and tangent loss (Figure 6d) as a function of the frequency at various ultrasonic amplitudes for the pure PP, devulcanized EPDM, and PP/EPDM blends of the ratios of 75/25, 50/50, and 25/75 from TSE using the DR method. In this case, the complex viscosity of the blends still increased with the increasing EPDM concentration, because the higher viscosity of EPDM rubber compared to that of PP caused the higher viscosity of the PP/EPDM blends. However, unlike the OS and TS methods, at all different ratios of the PP/EPDM blends the complex viscosity increased with amplitudes at 10 and 13 μm. The storage (Figure 6b) and loss (Figure 6c) moduli also increased dramatically at an ultrasonic amplitude of 13 μm. The loss tangent (Figure 6d) had the lowest value at an ultrasonic amplitude of 13 μm. According to a previous study [11], molecular transformations, such as the formation of a copolymer, might occur during the dynamic revulcanization in the extrusion simultaneously with the generation of the dense chemical network in the ultrasonically treated EPDM phase, leading to an increase in the viscosity. Additionally, the compatibilization of PP/EPDM and the good dispersion of EPDM particles in the blends might lead to an increase in viscosity [15]. The latter is caused by the fact that in the DR method the EPDM particles were cured during the extrusion, preventing their agglomeration due to their high viscosity and poor flowability. This results in the good dispersion of dynamically revulcanized EPDM particles in the PP phase. Therefore, the relative contribution of these effects defines the viscosity of these blends. These effects are strongest at an amplitude of 13 μm due to the higher level of devulcanization of EPDM rubber, as reflected by the reduced storage modulus of devulcanized EPDM rubber at the high amplitude.

**Figure 7:**
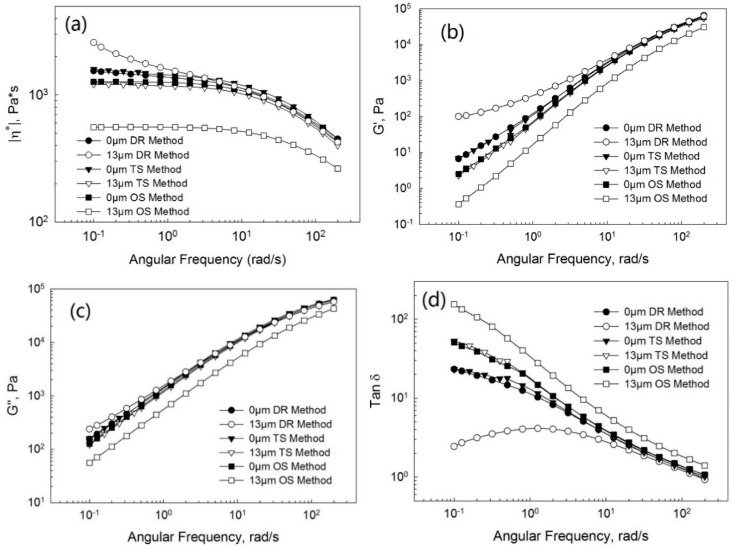
Complex viscosity (**a**), storage modulus (**b**), loss modulus (**c**), and loss tangent (**d**) as a function of the frequency at the ultrasonic amplitudes of 0 μm (without ultrasonic treatment) and 13 μm for the PP/EPDM 75/25 blends prepared by the OS, TS, and DR methods.

**Figure 8:**
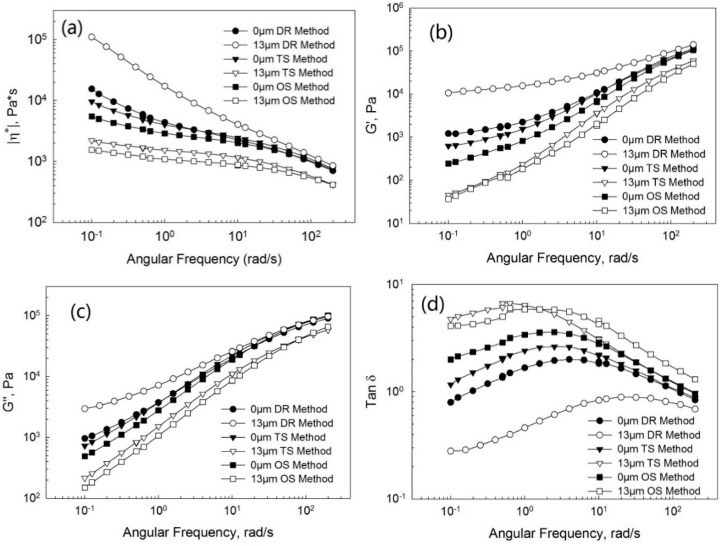
Complex viscosity (**a**), storage moduli (**b**), loss moduli (**c**), and loss tangent (**d**) as a function of the frequency at the ultrasonic amplitudes of 0 μm (without ultrasonic treatment) and 13 μm for the PP/EPDM 50/50 blends prepared by the OS, TS, and DR methods.

**Figure 9:**
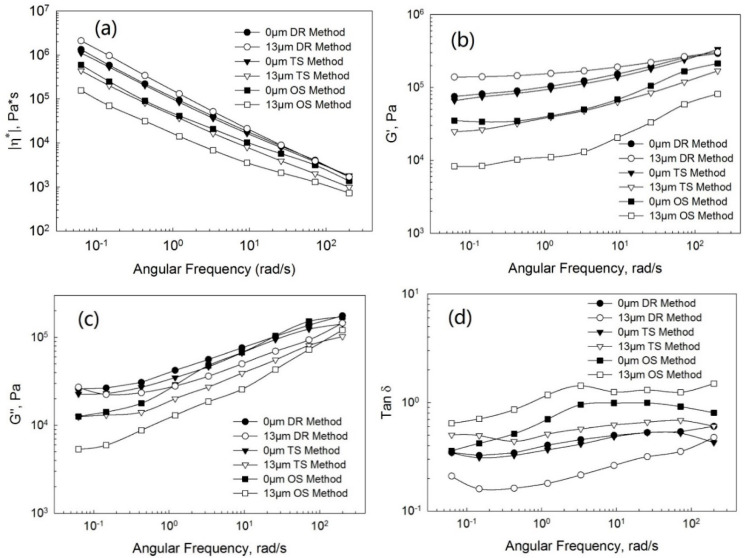
Complex viscosity (**a**), storage moduli (**b**), loss moduli (**c**), and loss tangent (**d**) as a function of the frequency at the ultrasonic amplitudes of 0 μm (without ultrasonic treatment) and 13 μm for the PP/EPDM 25/75 blends prepared by the OS, TS, and DR methods.

Figure 7, Figure 8 and Figure 9 show the complex viscosity (Figure 7a, Figure 8a and Figure 9a), storage (Figure 7b, Figure 8b and Figure 9b), and loss (Figure 7c, Figure 8c and Figure 9c) moduli and loss tangent (Figure 7d, Figure 8d and Figure 9d) as a function of the frequency for PP/EPDM blends of ratios of 75/25, 50/50, and 25/75 at the ultrasonic amplitudes of 0 μm (without ultrasonic treatment) and 13 μm from TSE using the OS, TS, and DR methods, respectively. Firstly, at an ultrasonic amplitude of 0 μm the complex viscosity, storage, and loss moduli of PP/EPDM blends using the DR method was only slightly higher than those of PP/EPDM blends using the TS method. The loss tangent of PP/EPDM blends using the DR method was lower than that of PP/EPDM blends using the TS method. It can be explained that the chemical network generated by curing in the EPDM phase of the dynamically revulcanized PP/EPDM blends obtained from the DR method led to the better compatibilization between PP and EPDM rubber, resulting in a higher modulus and complex viscosity and a lower loss tangent. The complex viscosity, storage, and loss moduli of PP/EPDM blends using the OS method were lower than those of the PP/EPDM blends using the DR and TS methods. The loss tangent of the PP/EPDM blends using the OS method was higher than that of the PP/EPDM blends using the DR and TS methods. Based on the previous study [14], this can be explained by the fact that compatibilization between PP and EPDM rubber using the DR and TS methods might be better than that in the OS method, resulting in the higher moduli and viscosity of PP/EPDM blends using the DR and TS methods.

Secondly, at an amplitude of 13 μm the complex viscosity, storage, and loss moduli of the PP/EPDM blends using the DR method were higher than those of the PP/EPDM blends using the TS method. The loss tangent of the PP/EPDM blends using the DR method was lower than that of the PP/EPDM blends using the TS method. Again, based on the previous study [15] it can be explained that molecular transformations such as the formation of a copolymer might occur during the dynamic revulcanization in the extrusion simultaneously with the generation of a dense chemical network in the curing of the EPDM phase in PP/EPDM blends using the DR method. Additionally, a higher level of compatibilization between PP and EPDM rubber using the DR method might lead to an increase in the modulus and viscosity of PP/EPDM blends in comparison with the TS method. Morphology study, shown later, shows that the dispersion of EPDM particles in the PP/EPDM blends using the DR method was much better than that of the PP/EPDM blends using the TS method. This may also contribute to the higher moduli and viscosity in the case of PP/EPDM blends in the DR method. The complex viscosity, storage, and loss moduli of the PP/EPDM blends using the TS method were higher and the loss tangent was lower than those of the PP/EPDM blends using the OS method. This is due to the degradation of PP in the PP/EPDM blends prepared by the OS method, but the degradation did not occur in the TS method because PP was not treated by the ultrasound.

### 3.3. Mechanical Properties

**Figure 10:**
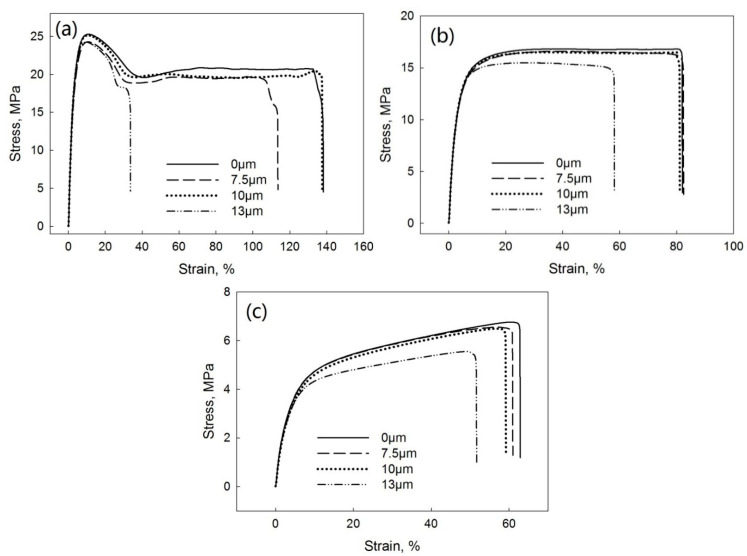
Stress–strain curves of PP/EPDM 75/25 (**a**), 50/50 (**b**), and 25/75 (**c**) blends prepared at different ultrasonic amplitudes by the OS method.

**Figure 11:**
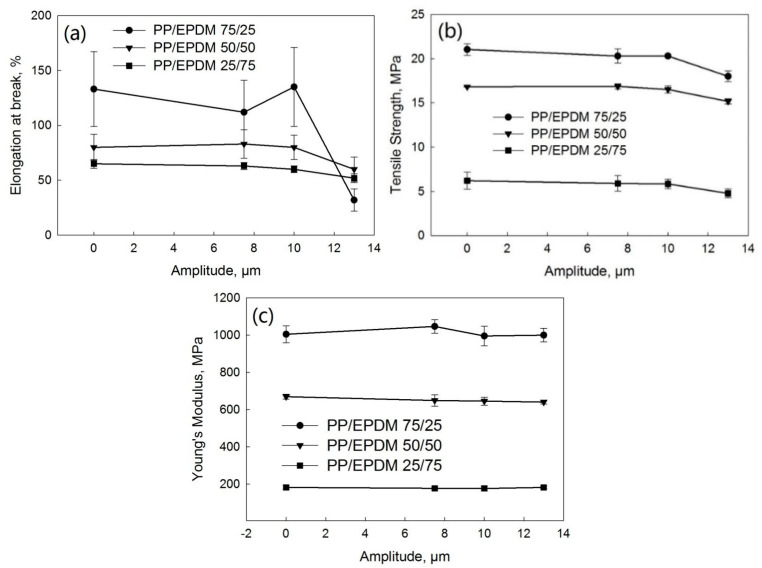
Elongation at break (**a**), tensile strength (**b**), and Young’s modulus (**c**) as a function of the ultrasonic amplitude for various ratios of the PP/EPDM blends prepared by the OS method.

Figure 10 shows the tensile stress–strain curves of PP/EPDM blends at ratios of 75/25 (Figure 10a), 50/50 (Figure 10b), and 25/75 (Figure 10c) manufactured at different ultrasonic amplitudes by the OS method. Figure 11 indicates the elongation at break (Figure 11a), tensile strength (Figure 11b), and Young’s modulus (Figure 11c) as a function of the ultrasonic amplitude for PP/EPDM blends of different ratios obtained by the OS method. The Young’s modulus at different ratios of PP/EPDM was not affected by an increase in the ultrasonic amplitude. The elongation at break and tensile strength decreased with an increase in the ultrasonic amplitude at different ratios of PP/EPDM, with the lowest value being at an amplitude of 13 μm. This is because the complex viscosity of the PP/EPDM blends was lowest at an amplitude of 13 μm (Figure 4) due to the coalescence of EPDM particles in the PP phase during the extrusion. The coalescence of EPDM particles led to agglomerates of EPDM phase in the PP, as supported by the morphology study shown later. The agglomeration led to the poor dispersion of the EPDM phase in the PP, resulting in poor mechanical properties. In an earlier study [17], PP/waste EPDM at a ratio of 25/75 was compounded in TSE with the aid of ultrasonic waves. The mechanical properties of the ultrasonically treated PP/waste EPDM decreased due to the low chemical interaction at the interface and the poor adhesion between the waste EPDM and PP.

**Figure 12:**
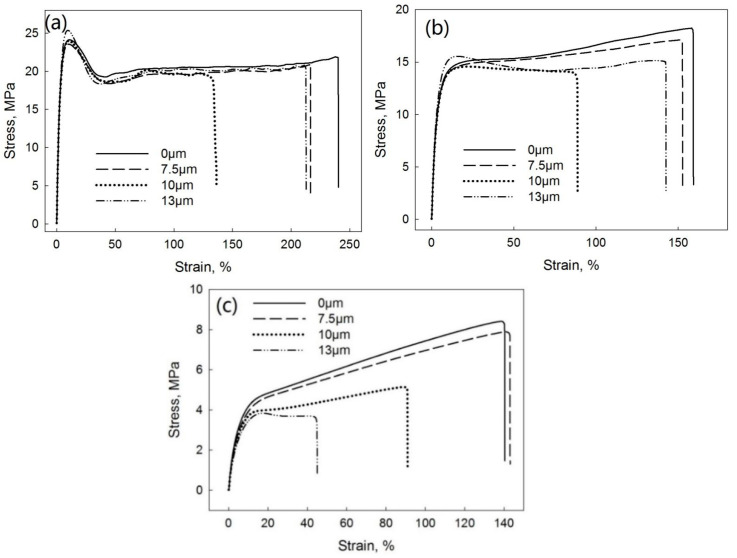
Stress–strain curves of PP/EPDM 75/25 (**a**), 50/50 (**b**), and 25/75 (**c**) blends prepared at different ultrasonic amplitudes by the TS method.

**Figure 13:**
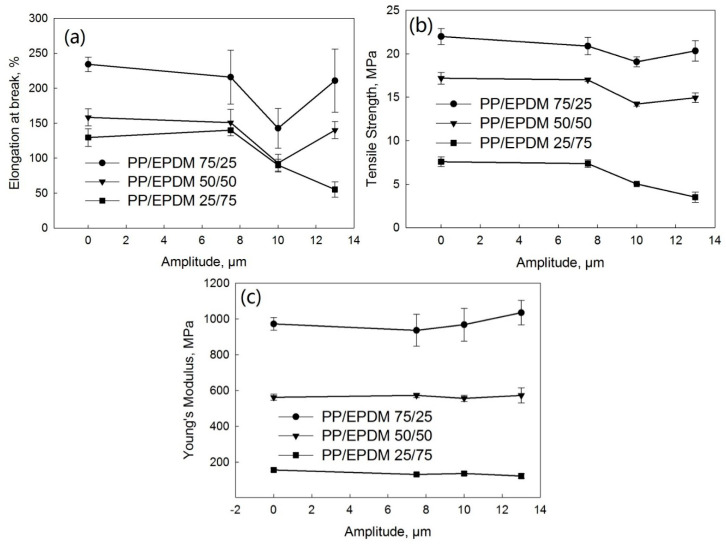
Elongation at break (**a**), tensile strength (**b**), and Young’s modulus (**c**) as a function of ultrasonic amplitude for PP/EPDM blends of different ratios prepared by the TS method.

Figure 12 shows the tensile stress–strain curves of PP/EPDM blends at ratios of 75/25 (Figure 12a), 50/50 (Figure 12b), and 25/75 (Figure 12c) manufactured at different ultrasonic amplitudes by the TS method. Figure 13 indicates the elongation at break (Figure 13a), tensile strength (Figure 13b), and Young’s modulus (Figure 13c) as a function of the ultrasonic amplitude for PP/EPDM blends of different ratios. As indicated in Figure 13, the modulus was unaffected by an increase in the ultrasonic amplitude at different ratios of PP/EPDM. The elongation at break and tensile strength were decreased for all the ultrasonically treated samples. This is because the complex viscosity of the PP/EPDM blends decreased for the treated samples, as shown in Figure 5, due to the coalescence of EPDM particles in the PP during the extrusion. The coalescence of the EPDM particles led to the agglomeration of EPDM, as shown later. The agglomeration led to the poor dispersion of EPDM particles in the PP/EPDM blends, resulting in their poor mechanical properties. In earlier study,^16^ PP was compounded with ultrasonically treated waste EPDM rubber at a ratio of 25/75. The size of original waste EPDM powder used was much smaller (5 to 20 μm) than that in the present study (150 to 250 μm). The smaller size of EPDM particles in the early study led to the good dispersion of EPDM particles in the PP phase, resulting in the higher elongation at break and higher tensile strength. However, in an early study [17] the elongation at break of the PP/waste EPDM blends decreased from 300% to 150% and the tensile strength decreased from 12 to 5 MPa with the increasing ultrasonic amplitude. This was explained to be due to the low chemical interaction at the interface and the poor adhesion between waste EPDM and PP. In the present study, the elongation at break of the PP/waste EPDM blends at a ratio of 25/75 decreased from 130% to 60% and the tensile strength decreased from 7 to 4 MPa with the increasing ultrasonic amplitude due to the agglomeration of EPDM particles in the blend.

**Figure 14:**
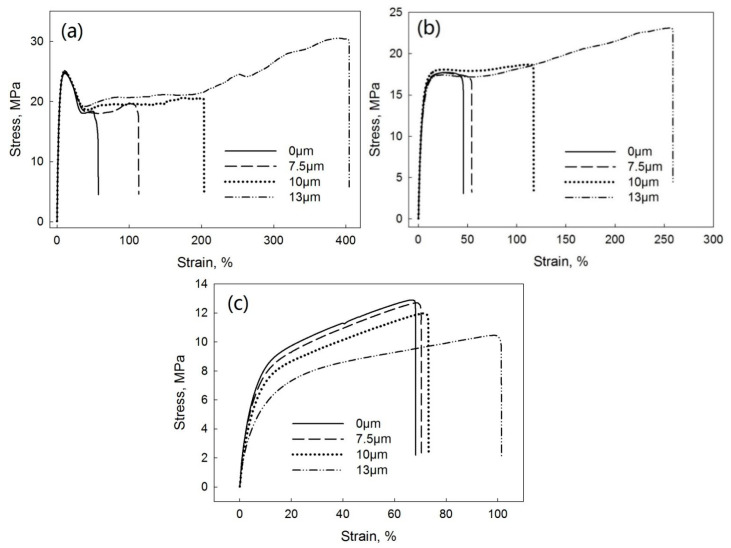
Stress–strain curves of PP/EPDM 75/25 (**a**), 50/50 (**b**), and 25/75 (**c**) blends prepared at different ultrasonic amplitudes by the DR method.

**Figure 15:**
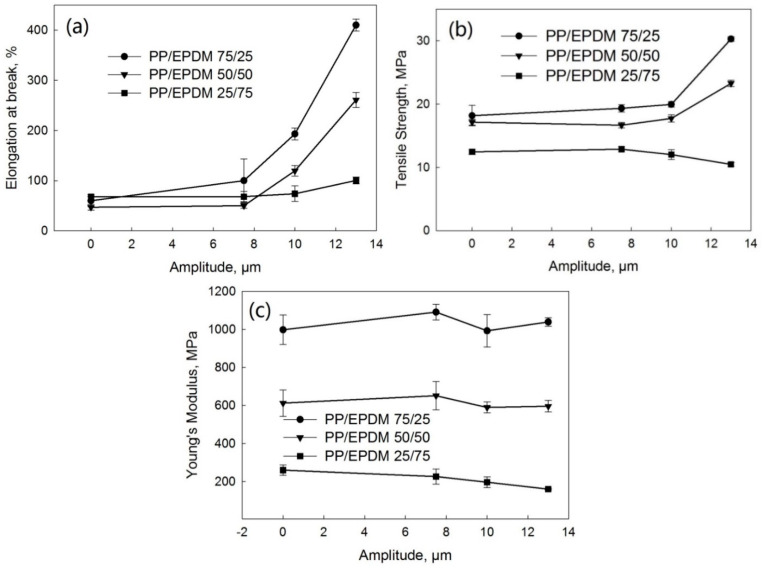
Elongation at break (**a**), tensile strength (**b**), and Young’s modulus (**c**) as a function of ultrasonic amplitude for PP/EPDM blends of various ratios prepared by the DR method.

Figure 14 shows the tensile stress–strain curves of PP/EPDM blends at ratios of 75/25 (Figure 14a), 50/50 (Figure 14b), and 25/75 (Figure 14c) manufactured at different ultrasonic amplitudes by the DR method. Figure 15 indicates the elongation at break (Figure 15a), tensile strength (Figure 15b), and Young’s modulus (Figure 15c) as a function of the ultrasonic amplitude for PP/EPDM blends of different ratios. The elongation at break of PP/EPDM blends at different ratios increased with increasing ultrasonic amplitude. Clearly, this increase in the elongation at break in PP/EPDM blends obtained in the DR method is an indication of their increased elasticity. Additionally, the tensile strength of PP/EPDM blends at ratios of 75/25 and 50/50 increased with the increasing ultrasonic amplitude. Based on the previous study [16], molecular transformations such as the formation of a copolymer and the generation of the dense chemical network in the ultrasonically treated EPDM phase occur during the dynamic revulcanization in the extrusion process. It is believed that the copolymer was formed at the interface of two phases, reducing the interfacial tension between the two components in blends and improving their mechanical properties. This compatibilization between PP and EPDM rubber led to the good dispersion and smaller sizes of EPDM particles in the PP continuous phase during dynamic revulcanization, as shown below by the morphological study. This is because in the DR method the EPDM particles were cured during the extrusion, preventing their agglomeration due to their high viscosity and poor flowability. This resulted in the good dispersion of EPDM particles in the PP phase. Additionally, for PP/EPDM blends at a ratio of 25/75, the tensile strength and Young’s modulus slightly decreased, while the elongation at break slightly increased with the increasing ultrasonic amplitude. This is due to the lower gel fraction of devulcanized EPDM rubber at an amplitude of 13 μm.

### 3.4. Morphology of Blends

**Figure 16:**
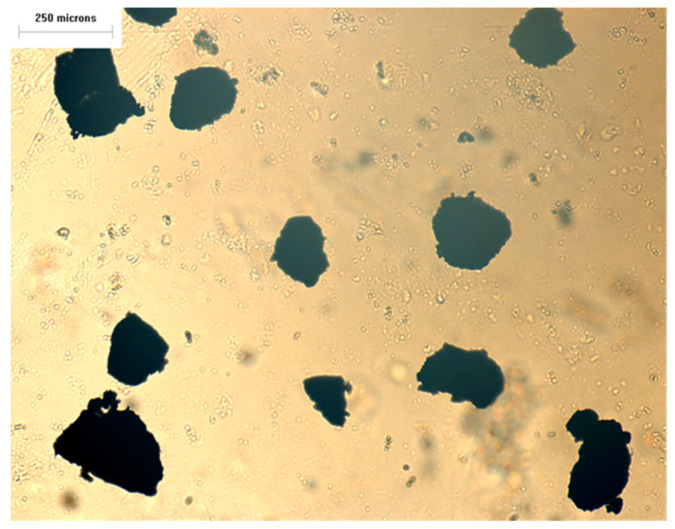
Optical micrographs of the original EPDM powder.

Figure 16 shows the micrograph of original 40-mesh waste EPDM powder as obtained by the optical transmission microscopy. It indicates that diameters of the EPDM powder range from 100 to 250 μm.

**Figure 17:**
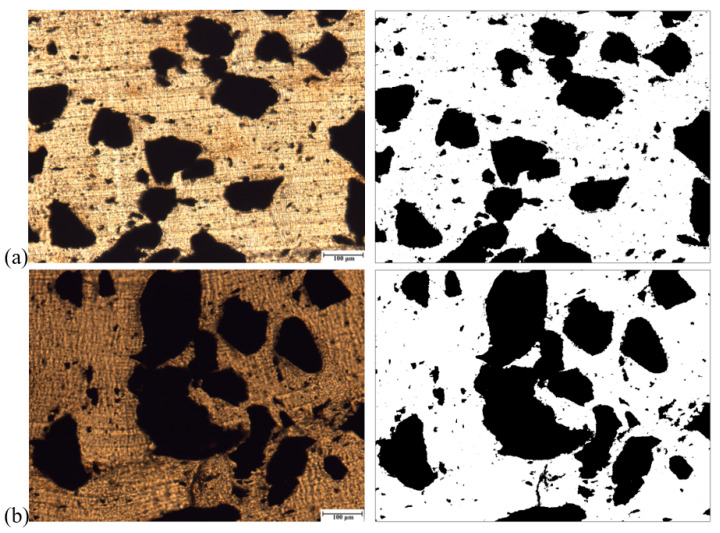
Optical micrographs of PP/EPDM 75/25 blends without (**a**) and with ultrasonic treatment at an amplitude of 13 μm (**b**) prepared by the OS method (right micrographs were treated by the ImageJ software).

**Figure 18:**
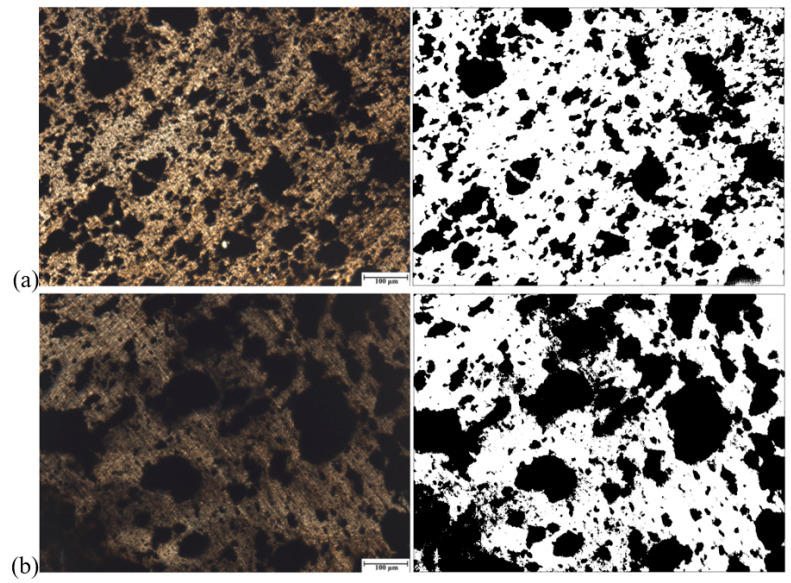
Optical micrographs of PP/EPDM 75/25 blends without (**a**) and with ultrasonic treatment at an amplitude of 13 μm (**b**) prepared by the TS method (right micrographs were treated by the ImageJ software).

**Figure 19:**
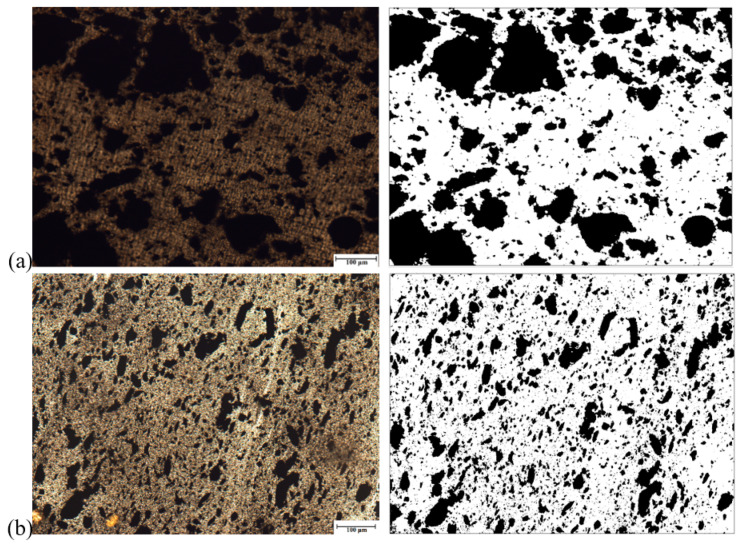
Optical micrographs of PP/EPDM 75/25 blends without (**a**) and with ultrasonic treatment at an amplitude of 13 μm (**b**) prepared by the DR method (Right micrographs were treated by the ImageJ software).

Figure 17 shows the SEM micrographs of the 75/25 PP/EPDM blends obtained using the OS method without (Figure 17a) and with ultrasonic treatment at an amplitude of 13 μm (Figure 17b). Firstly, it can be observed from Figure 17 that the particles in blends are much smaller than those of the original EPDM powder. This is because of the fact that the EPDM rubber was devulcanized during the ultrasonic extrusion. It is noteworthy that there are some agglomerates of EPDM particles in the 75/25 PP/EPDM blend treated at an amplitude of 13 μm. This is due to the fact that, at such a high ultrasonic amplitudes, devulcanized EPDM particles have a lower viscosity, leading to their coalescence (agglomeration) during the extrusion. The formation of the agglomerates led to poor mechanical properties, as was shown earlier for these blends. The similar effect is observed for the 75/25 PP/EPDM blend obtained using the TS method without treatment and with ultrasonic treatment at an amplitude of 13 μm. This is seen in Figure 18a,b. The optical micrographs of the 75/25 PP/EPDM blends obtained using the DR method without treatment (Figure 18a) and with ultrasonic treatment at an amplitude of 13 μm (Figure 18b) are presented in Figure 19. One can observe in Figure 19b that the EPDM particles in the blends obtained at an amplitude of 13 μm exhibit much smaller sizes and a better dispersion. This is because in the DR method the EPDM particles were dynamically revulcanized during the extrusion, preventing their agglomeration due to their high viscosity and poor flowability. The latter leads to a smaller size of EPDM particles and their good dispersion in the PP matrix. These are reasons why the best improvement in the mechanical properties of the PP/EPDM blends was obtained using the DR method.

**Figure 20:**
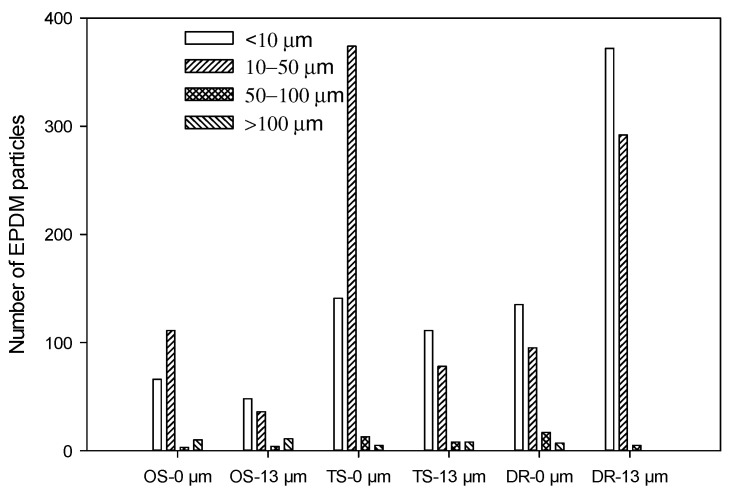
Analysis of the EPDM particle number in PP/EPDM 75/25 blends using different methods.

The distribution of EPDM particles sizes in the PP/EPDM 75/25 blends obtained by the OS, TS, and DR methods without treatment and with ultrasonic treatment at an amplitude of 13 μm is given in Figure 20. It can be seen that the blend prepared using the OS method exhibits the largest number of EPDM particles of sizes of over 100 μm, while the blend prepared using the DR method exhibits the lowest number of large particles. In fact, the blend obtained by the DR method at an amplitude of 13 μm does not contain EPDM particles over 100 μm. Additionally, it contains the largest number of EPDM particles with sizes less than 10 μm. The analysis presented in Figure 20 correlates well with the rheological and mechanical properties discussed earlier.

## 4. Conclusions

Compounding PP with waste EPDM using ultrasonically aided extrusion was carried out. The effect of the PP/waste EPDM ratio, compounding method, and ultrasonic amplitude were investigated. The one-step (OS), two-step (TS), and dynamic revulcanization (DR) methods were applied using an ultrasonic TSE at ultrasonic amplitudes varying from 0 to 13 μm. PP and waste EPDM were compounded at ratios of 75/25, 50/50, and 25/75. In the OS method, PP and waste EPDM particles were compounded in the extruder with and without ultrasonic treatment. In the TS and DR methods, the waste EPDM particles were fed into the extruder and devulcanized without and with ultrasonic treatment. Then, in the TS method devulcanized EPDM was compounded with PP in the extruder without the imposition of ultrasound. In the DR method, devulcanized EPDM after compounding with curatives was mixed with PP and dynamically revulcanized in the extruder without the imposition of ultrasound. It was found that in the DR method, the mechanical properties of the PP/EPDM blends increased with the increase in the ultrasonic amplitude due to the formation of copolymer, the compatibilization between PP and EPDM rubber, and the good dispersion of revulcanized EPDM particles in the PP matrix phase. In particular, the tensile strength of 30 MPa and the elongation at break of 400% were achieved at an amplitude of 13 μm for the PP/EPDM blend at ratio of 75/25. However, in the OS and TS methods, the mechanical properties of the PP/EPDM blends decreased with an increase in the ultrasonic amplitude due to the coalescence of devulcanized EPDM particles, leading to the creation of agglomerates of EPDM particles in the PP phase. These findings are supported by optical microscopy studies of various blends. In the DR method, the complex viscosity increases with increasing ultrasonic amplitude due to the presence of the dense network of EPDM phase in the PP/EPDM blends and the good dispersion of EPDM particles in the PP phase. In the OS method, the complex viscosity decreases with the ultrasonic amplitude due to the degradation of PP and the devulcanization of EPDM particles. In the TS method, the complex viscosity decreases with the ultrasonic amplitude due to the devulcanization of EPDM particles.

## Data Availability

Data are in the authors possession.
